# Uncertainties of Economic Policy and Government Management Stability Played Important Roles in Increasing Suicides in Japan from 2009 to 2023

**DOI:** 10.3390/ijerph21101366

**Published:** 2024-10-16

**Authors:** Ruri Okubo, Ryusuke Matsumoto, Eishi Motomura, Motohiro Okada

**Affiliations:** Department of Neuropsychiatry, Division of Neuroscience, Graduate School of Medicine, Mie University, Tsu 514-8507, Japan; okubo-r@med.mie-u.ac.jp (R.O.); matsumoto-r@clin.medic.mie-u.ac.jp (R.M.); motomura@clin.medic.mie-u.ac.jp (E.M.)

**Keywords:** suicide, COVID-19, Japan, uncertainty, social standing, General Principles of Suicide Prevention Policy

## Abstract

Standardized suicide mortality rates per 100,000 (SMRs) in Japan consistently decreased from 2009 to 2019 but increased from 2020. The causes of these temporal SMR fluctuations remain to be clarified. Therefore, this study was conducted to identify the causalities underlying the recently transformed fluctuations of suicide mortality in Japan. Monthly suicide numbers disaggregated by sex and social standing, and political uncertainty indices, such as economic policy uncertainty (EPU) and government management instability (AENROP), were obtained from Japanese government databases. Interrupted time-series analysis was performed to analyze temporal fluctuations of SMRs disaggregated by sex/social standing associated with the three General Principles of Suicide Prevention Policy (GPSPP) periods and the COVID-19 pandemic. Panel data and vector autoregressive analyses were conducted to investigate causalities from political uncertainties to SMRs. During the first and second GPSPPs (2009–2017), all SMRs disaggregated by sex and social standing decreased, whereas those of unemployed females did not change. During the third GPSPP (2017–2022), decreasing trends in all SMRs were attenuated compared to previous periods. All female SMRs, except unemployed females, showed sharp increases synchronized with the pandemic outbreak. No male SMRs showed sharply increasing at the pandemic outbreak. SMRs of unemployed males/females drastically increased in the later periods of the pandemic, while SMRs of employed and multiple-person/single-person household males did not increase during the pandemic. SMR of unemployed males was positively related to AENROP but not EPU. Other male SMRs were positively related to EPU/AENROP. On the contrary, not all female SMRs were related to EPU/AENROP. Increasing AENROP generally contributed to increasing male SMRs throughout the observation period; however, susceptibility to AENROP and/or political information might have unexpectedly contributed to suppressing the sharply increasing male SMRs induced by large-scale social shocks (the COVID-19 pandemic outbreak) in Japan.

## 1. Introduction

Japan successfully decreased standardized suicide mortality rates per 100,000 population (SMR) by approximately 30% from 2009 to 2019; however, SMRs in Japan began increasing from mid-2020 [1,2,3,4,5,6,7,8,9,10,11,12,13]. The COVID-19 pandemic is believed to play an important role in the cause of recent increasing SMRs in Japan due to temporal synchronization between the pandemic outbreak and increasing SMR [4,11,14]. However, the increasing SMRs in Japan during the pandemic are exceptional internationally, as the majority of OECD countries (other than South Korea, Ireland, Hungary and Spain) experienced decreasing or unchanging suicide during the pandemic in comparison to before the pandemic outbreak [3,15,16,17,18,19,20,21]. Several time-series analyses using seasonal auto-regressive integrated moving average (sARIMA), joinpoint regression or interrupted time-series analysis (ITSA) have identified two temporal fluctuation patterns in SMRs in Japan where the groups at risk for suicide (males < 30 years and females < 50 years of ages) during the pandemic sharply increased in synchrony with the pandemic outbreak and the attenuation of decreasing trends from 2017 was also observed [14,22,23,24,25,26].

These findings suggest that the recent increase in SMRs in Japan is possibly caused by the complicated interaction between factors associated with the pandemic and other factors that affected lives before the pandemic [14,22,23,24,25,26,27,28,29]. However, in spite of these efforts, the actual causes underlying the increasing SMRs in Japan since 2020 remain to be clarified. It has been concerned that ITSA might overestimate the sharply increasing SMRs synchronized with the COVID-19 pandemic outbreak affected by the attenuation of decreasing trends in SMRs from 2017 [25,26]. It is noteworthy that the General Principles of Suicide Prevention Policy (GPSPP) was updated from the second to the third version in July 2017 [30]. The GPSPP, which provides guidelines for comprehensive suicide prevention programs (Supplementary Table S1), has been shown to play roles in consistently decreasing SMRs in Japan through financial support, enhancing regional welfare and social safety nets, and improving regional vulnerabilities in social protection across communities, workplaces, and schools [13,28]. Therefore, to detect the actual SMR fluctuation using ITSA, it is necessary to add GPSPP periods to the pandemic outbreak as the intervention periods [28].

“Individuals experienced socioeconomic and psychosocial deterioration during the pandemic due to social restrictions, including governmental restriction measures and self-regulatory restrictions, and the impairment of traditional communities in regions, workplaces and schools” [31,32,33,34,35,36], Durkheim’s anomie theory suggests that social situations like these can disrupt the normative integration of individuals and lead to anomic shocks, which can trigger anomic suicides [37,38]. The sharply increasing SMRs in Japan, synchronized with the COVID-19 pandemic, are consistent with the characteristics of anomie suicide [24,25]. Although the SMRs of working-age females and unemployment rates have similar fluctuation patterns, the impact of socioeconomic deterioration was reported to be smaller than expected [22,23,24,25]. A statistical analysis using independent variables, such as social factors associated with anomic shock, may identify some of the causes underlying the increasing SMRs that were observed after the pandemic outbreak in Japan. Recent studies have reported that economic policy uncertainty and government management instability played important roles in increasing SMRs both globally and in some countries, including Japan [39,40,41,42,43].

Based on these backgrounds, to identify the features of recent increasing suicides in Japan, this study conducted the following two statistical processes: (1) the temporal fluctuations of age-standardized suicide death rates (SDRs) disaggregated by sex (males and females) and SMRs disaggregated by social standing (multiple-person household residents, single-person household residents, employed individuals and unemployed individuals) using an ITSA set at GPSPP periods and the COVID-19 pandemic outbreak as intervention periods, and (2) a hierarchical linear regression (HLM) and vector autoregressive analysis (VAR), which were used to detect the impacts of indices of economic policy uncertainty and government management instability on SDRs and SMRs.

## 2. Materials and Methods

### 2.1. Ethical Considerations and Reporting Guidelines

This study adhered to the Strengthening the Reporting of Observational Studies in Epidemiology (STROBE) Reporting Guidelines. The Medical Ethics Review Committee of Mie University waived the requirements for informed consent and ethical approval because the study used data available from publicly accessible governmental databases.

### 2.2. Data Source

Monthly suicide data disaggregated by prefecture, sex and social standing (multiple-person/single-person household residents and employed/unemployed individuals) were obtained from Basic Data on Suicide in the Region published by the Ministry of Health, Labor and Welfare (MHLW). The Japanese government provides two national suicide databases, including Basic Data on Suicide in the Region, which is recompiled Suicide Statistics (SSNPA) managed by the National Police Agency and Vital Statistics Registration managed by the MHLW [44,45]. Basic Data on Suicide in the Region and SSNPA by MHLW provide detailed suicide statistics for planning GPSPP and have been published by MHLW since January 2009 [46]. SSNAP has been evaluated to be the internationally most accurate and rapidly published government suicide statistics since SSNPA published the monthly suicide numbers disaggregated by various factors, which were investigated by judicial police in each region via conducting physiological examinations and searching the personal characteristics and backgrounds of each suicide case, within the next month [24,25,28,29].

In Japan, only medical doctors can prepare death certificates, and the Medical Practitioners Law stipulates that abnormal deaths must be reported to the National Police Agency within 24 h. The National Police Agency must conduct physiological examinations to determine the course of death in all cases with an abnormal cause of death [28,29]. As it is impossible to collect suicide motives from the victims themselves, to eliminate subjectivity as much as possible, the police investigate suicide motives based on evidence, including suicide notes, official documentation (i.e., medical certificates and clinical recordings) and testimony from the victim’s family. The results of this investigation suggest different motives for suicide, and these motives are compared to previously compiled lists of motives. In the SSNPA, suicides are classified into seven major categories: health-, family-, economic-, romance-, employment-, school-related problems, and others (including 52 subcategories). Detailed explanations of the suicide motives have been described in previous reports [24,28,29]. The Vital Statistics Registration is a cause-of-death statistics database that publishes suicide, homicide, accidental, and unexplained deaths. The Vital Statistics Registration published the annual number of suicides disaggregated by age in 5-year intervals (15–19 years and 20–24 years). However, the Vital Statistics Registration does not provide suicide numbers disaggregated by social standing. Therefore, the present study adopted the suicide statistics in Basic Data on Suicide in the Region for analysis.

Populations disaggregated by prefecture, sex, and age were obtained from ‘Regional Statistics of the System of Social and Demographic’ published in e-Stat. Populations of single-person and multiple-person household residents disaggregated by prefecture and sex were obtained from the Comprehensive Survey of Living Conditions of MHLW. The population of employed and unemployed individuals was obtained from the Labor Force Survey of MHLW.

Monthly indices of economic policy uncertainty (EPU) and government management instability (AENROP) (normalized to average values of 100 in 1987–2015) were obtained from the Research Institute of Economy, Trade, and Industry (RIETI) (Tokyo, Japan) [47,48]. Most established an EPU index globally, and 23 countries, including Japan, had their Index developed by Baker and his colleagues [49] based on text mining using newspapers to capture policy-related economic uncertainty, the number of federal tax code provisions in a country, and disagreement among economic forecasters [49]. Based on Baker’s concept, Japan’s EPU is devised from four major newspapers (Yomiuri, Asahi, Mainichi, and Nikkei) [50]. The term sets used to derive the EPU index are described in Supplementary Table S2. The AENROP index was developed to capture the uncertainty of stability of government management in Japan, using political party support ratings in regular monthly opinion polls from news agencies (Jiji Press, Kyodo News), newspapers (Yomiuri, Asahi, Mainichi, Nikkei) and television station networks (NHK, NNN and JNN) by RIETI [47,48]. Higher EPU and AENROP indices indicated increasing uncertainty [48,50]. The observation period was set between January 2009 (when Basic Data on Suicide in the Region began providing monthly suicide numbers) and June 2023 (the end of the COVID-19 pandemic) [51].

### 2.3. Data Analysis

Monthly SMRs were calculated by dividing the monthly suicide numbers by the 100,000 populationof the corresponding groups in the same period, disaggregated by sex and social standing. The monthly SMRs were then converted to annualized values for 365 days. Age-standardized suicide death rates (SDRs) of males and females in Japan were adjusted using the Japanese standard population distribution model in 2015 (2015JSP) [52].

ITSA with robust standard errors was used to analyze temporal fluctuations of SDRs and SMRs disaggregated by sex and social standing, including trends and discontinuity and their effect size using Stata version 17 for Windows (StataCorp, College Station, TX, USA) [25,28,53]. The intervention periods in the ITSA were set to August 2012, July 2017, and April 2020, based on the periods of the 1st GPSPP (June 2007 to August 2012), 2nd GPSPP (August 2012 to July 2017), 3rd GPSPP (July 2017 to October 2022) and the COVID-19 pandemic outbreak (April 2020) [14,30]. The detailed Stata codes for ITSA have been previously described [25,28,53].

The temporal causality from EPU and AENROP to SDRs and SMRs was analyzed using vector autoregressive analysis with Granger causality and robust standard errors (VAR) using Gretl for Windows v2023c (https://gretl.sourceforge.net/win32/index_es.html, accessed on 1 January 2024.) [26]. When the assumption of Granger causality was violated (*p* < 0.05), sensitivity analyses were conducted using forecast variance decomposition and impulse response analyses. The impacts of EPU and AENROP on SDRs and SMRs were determined using the hierarchical linear regression model with robust standard error (HLM) to control for variation among prefectures using Gretl v2023c.

## 3. Results

The descriptive statistics of the variables used for SMRs among males and females from January 2009 to June 2023 are indicated in Table 1.

The mean ± SD of SDRs and SMRs disaggregated by the social standing of males/females were as follows: SDRs, 27.1 ± 5.8/11.7 ± 2.2; employed individuals, 20.0 ± 4.4/5.3 ± 1.1; unemployed individuals, 70.3 ± 21.5/14.4 ± 6.9; multiple-person household residents, 19.5 ± 4.6/9.7 ± 2.0; and single-person household residents, 85.3 ± 20.0/25.6 ± 5.8.

### 3.1. Temporal Fluctuations of SDRs and Uncertainty Indices

**Figure 1:**
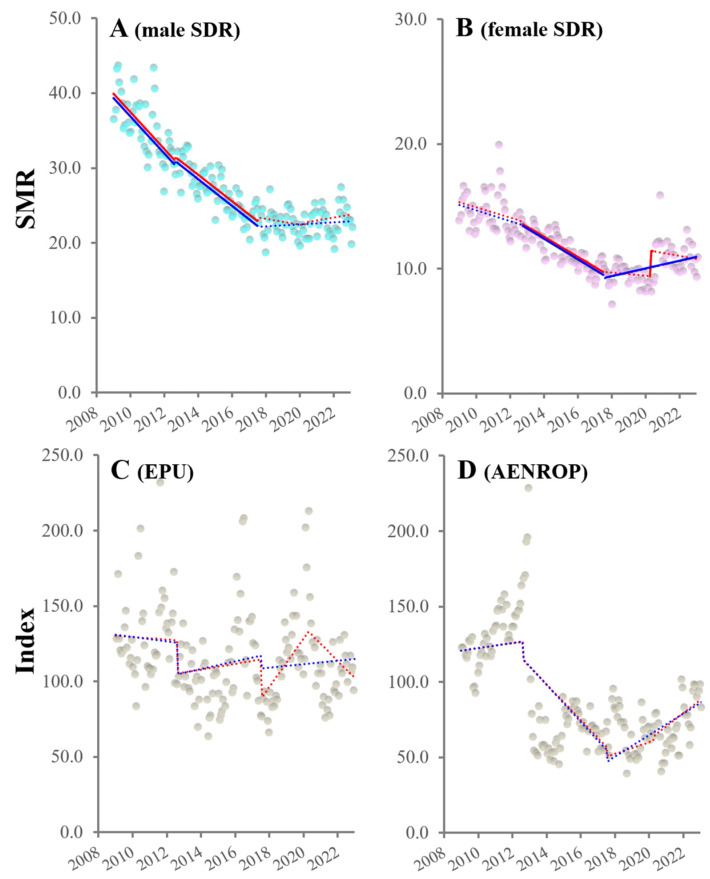
Fluctuation in SDRs and uncertainty indices (EPU: economic policy uncertainty, and AENROP: government management instability) from January 2009 to June 2023 in Japan. Panels (**A**–**D**) indicated the trends and discontinuity of SDRs among males and females, EPU and AENROP, from January 2009 to June 2023 in Japan, respectively. Ordinates indicate the SDR (per 100,000 in the population) in panels (**A**,**B**) and EPU and AENROP indices in panels (**C**,**D**). Blue and red circles indicate the observed annualized monthly SDRs of males and females, respectively. Grey circles indicate the observed uncertainty indices value. Blue and red lines indicate the results calculated by ITSA with interventions from three GPSPP periods alone and GPSPP periods alongside the COVID-19 pandemic outbreak, respectively. Solid and dotted lines indicate the significant and non-significant trends or discontinuity detected by ITSA, respectively.

When intervention periods in ITSA were set as GPSPP periods alone, male SDRs decreased during the first and second GPSPPs, but were unchanged during the third GPSPP. In contrast, female SDRs remained unchanged, decreased, and unchanged during the first GPSPP, second GPSPP and third GPSPPs, respectively (Figure 1). When the COVID-19 pandemic outbreak was added to the GPSPP periods as the intervention periods in ITSA, the ITSA detected no significant impact of the pandemic on the male SDR in the third GPSPP. The female SDR showed a sharp increase (positive discontinuity) that was synchronized with the pandemic outbreak; however, the trends remained unchanged both before and after the pandemic outbreak (Figure 1).

Unlike SDRs, the uncertainty indices (EPU and AENROP) did not show significant trends or discontinuity during the observed periods (Figure 1). From January 2009 to June 2023, the mean ± SD of EPU and AENROP were 116.1 ± 29.4 and 87.9 ± 35.3, respectively.

### 3.2. Temporal Fluctuations of SMRs Disaggregated by Social Standing

**Figure 2:**
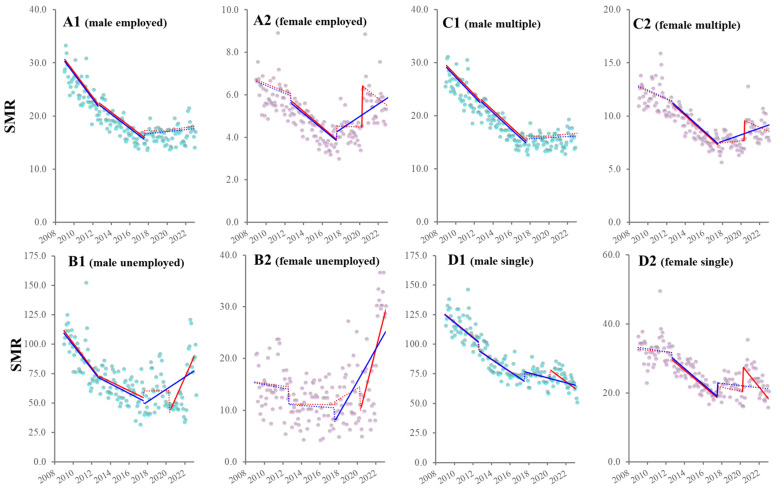
Fluctuation in SMRs disaggregated by social standing from January 2009 to June 2023 in Japan. Panels (**A**–**D**) indicate the trends and discontinuity of SMRs of employed, unemployed individuals, multiple-person and single-person household residents from January 2009 to June 2023 in Japan, respectively. Panels (**A1**–**D1**) and (**A2**–**D2**) indicate male and female SMRs disaggregated by social standing, respectively. Ordinate and abscissa indicate the SMR (per 100,000 population) and years, respectively. Blue and red circles indicate the observed annualized monthly SMRs of males and females, respectively. Blue and red lines indicate the results calculated by ITSA with interventions of GPSPP alone and GPSPP with the COVID-19 pandemic outbreak, respectively. Solid and dotted lines indicate the significant and non-significant trends or discontinuity of SMRs detected by ITSA, respectively.

When the interventions in ITSA were set to the three GPSPP periods alone, ITSA detected that all male SMRs of employed/unemployed individuals and multiple-person/single-person household residents decreased during the first and second GPSPPs. In contrast, during the third GPSPP, male SMRs of employed individuals and multiple-person household residents did not change, whereas those of unemployed individuals and single-person household residents increased and decreased, respectively (Figure 2). In females, during the first GPSPP, all SMRs disaggregated by social standing did not show significant changes. During the second GPSPP, the SMRs of employed females and multiple-person/single-person household residents decreased, but the SMR of unemployed females did not change. During the third GPSPP, female SMRs among employed/unemployed individuals and multiple-person household residents increased, while those of single-person household residents remained unchanged (Figure 2).

When the COVID-19 pandemic outbreak was added to the GPSPP periods for the intervention period in ITSA, the ITSA did not detect significant impacts of the pandemic on male SMRs in employed individuals or multiple-person household residents. In contrast, SMRs of unemployed individuals and single-person household residents remained unchanged before the pandemic outbreak in the third GPSPP; however, after the pandemic outbreak, trends in the SMRs of these groups transformed by increasing and decreasing, respectively (Figure 2). Sharp increases in all-male SMRs and the SMR of unemployed females synchronized with the pandemic outbreak were not observed; however, the female SMRs of employed individuals and multiple-person/single-person household residents showed sharp increases synchronized with the pandemic outbreak. Additionally, SMR trends in employed and multiple-person household females remained unchanged before and after the pandemic outbreak. Trends in female SMRs of unemployed individuals and single-person household residents transformed from unchanged before the pandemic outbreak to increasing and decreasing after the outbreak, respectively (Figure 2).

### 3.3. Causalities from Uncertainty Index for SDR/SMRs

#### 3.3.1. Fixed Effects of EPU and AENROP on SDR/SMRs

HLM detected positive fixed effects of EPU on the male SDR but not the female SDR (Table 2). However, HLM detected the positive fixed effects of EPU on male SMRs of employed individuals and multiple-person/single-person household residents, but EPU showed no relationship with male unemployed SMRs or all female SMRs disaggregated by social standing (Table 2). Contrary to males, the significant fixed effects of EPU on female SDRs and SMRs disaggregated by social standing (employed, unemployed individuals, multiple-person and single-person household residents) could not be detected (Table 2).

When AENROP was added to EPU as an independent variable, HLM detected positive fixed effects of AENROP on the male SDR, but those of EPU disappeared, whereas neither AENROP nor EUP had fixed effects on the female SDR (Table 3). Neither AENROP nor EUP showed fixed effects on any of the female SMRs (Table 3). Therefore, the HLM analysis suggested that increasing EPU and AENROP contributed to increasing male suicides but did not affect female suicides; however, the positive effect of government management instability was much stronger than that of economic policy uncertainty.

#### 3.3.2. Temporal Causalities from EPU and AENROP to SDR/SMRs

VAR detected positive temporal impacts of AENROP on the male SDR, similar to HLM, but did not detect a significant relationship with EPU. Neither AENROP nor EPU significantly affected the female SDR (Table 4). VAR also detected the positive temporal impacts of AENROP on all male SMRs disaggregated by social standing, while EPU did not show a temporal relationship. Neither AENROP nor EPU significantly affected any of the all-female SMRs disaggregated by social standing (Table 4).

**Figure 3:**
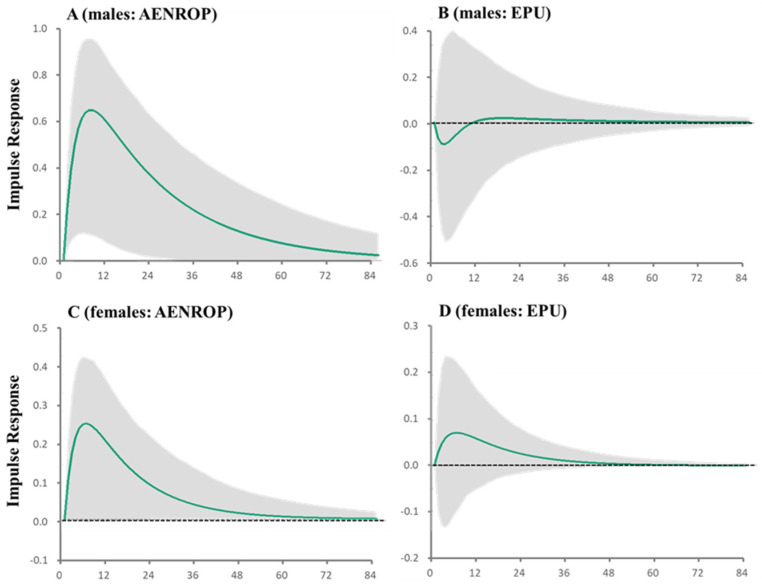
Impulse responses of SDRs in males and females to AENROP and EPU indices. Impulse responses of SDRs in males (**A**,**B**) and females (**C**,**D**) to increasing one standard deviation (SD) of AENROP (**A**,**C**) and EPU (**B**,**D**) indices. Green lines and grey regions indicate the mean ± 95% confidence interval (CI) of responses, respectively.

The impulse responses showed that the maximal impact of increasing AENROP on male SDRs was observed after six months, and its positive impact persisted for over two years (Figure 3).

## 4. Discussion

The present study demonstrates several specific features of SDRs in males and females, and SMRs disaggregated by sex and social standing (employed/unemployed individuals and multiple-person/single-person household residents), including temporal fluctuation and causalities from uncertainty indices (AENROP and EPU), in Japan from January 2009 to June 2023 (end of the COVID-19 pandemic). Suicide risks of unemployed and single-person household individuals were higher than those of unemployed and multiple-person household individuals in both males and females than employed individuals, respectively. The SMRs of unemployed individuals showed specific patterns of temporal fluctuations that were clearly different from those of the other groups. All male SMRs of employed/unemployed individuals and multiple-person/single-person household residents decreased during the first and second GPSPPs; however, trends in these SMRs transformed from unchanged before the pandemic to the third GPSPP. In contrast to males, none of the female SMRs significantly decreased during the first GPSPP or before the pandemic in the third GPSPP; however, during the second GPSPP, the SMRs of employed and multiple-person/single-person household females decreased, but those of unemployed females remained unchanged. The SMRs of employed and multiple-person/single-person household females sharply increased in synchrony with the COVID-19 pandemic outbreak, while those of males did not show a sharp increase. Interestingly, the SMRs of unemployed males and females did not indicate a sharply increasing synchrony with the pandemic outbreak but drastically increased after the outbreak, whereas, conversely, the SMRs of single-person household males and females decreased during the pandemic. These SMRs recovered to almost the equivalent of before the pandemic. Additionally, there were differences in the impacts of uncertainty indices (AENROP and EPU) on SDR/SMRs between males and females. AENROP was positively related to all male SMRs disaggregated by social standing, whereas EPU, on the other hand, was positively related to the male SMRs of employed individuals and multiple-person/single-person household residents but not to the SMR of unemployed males. Importantly, AENROP had a greater positive impact on male SMRs than EPU. In contrast, not all female SDRs or SMRs disaggregated by social standing were related to EPU or AENROP.

### 4.1. Impacts of Political Implementations on Suicide

The period of the second GPSPP was socially stable in comparison to the first and third GPSPPs, without large-scale disasters or economic shocks, and all SDRs and SMRs decreased in both males and females, with the exception of unemployed females. However, after the transition to the third GPSPP, trends in most SDRs and SMRs transformed from decreasing (in the second GPSPP) to unchanging (before the pandemic outbreak in the third GPSPP). We must note that the attenuation of decreasing SDR and SMR trends was independent of the pandemic since the decreasing trends in the third GPSPP were attenuated before the pandemic. This study could detect the attenuation of SDR and SMR trends due to switching from the second to the third GPSPP but could not analyze the causality underlying this attenuation in detail. The GPSPP is revised approximately every five years according to the Plan–Do–Check–Act cycle [28]. Initially, the first GPSPP listed 47 priorities in nine major priority categories, whereas the listed priorities decreased to 41 in the same nine major priority categories in the second GPSPP [30]. In contrast, the third GPSPP added several priorities, such as ‘decreasing SMRs of children/adolescents and socially vulnerable individuals’ and ‘strengthening organic collaboration among various suicide prevention programs completed through past GPSPPs to conventional suicide prevention programs,’ resulting in 67 priorities in twelve major priority categories [30]. These are additions rather than revisions or improvements to priority measures, and no factors seem to suppress the decreasing SDR and SMR trends. Several studies on the cost-effectiveness of the first and second GPSPPs reported that suicide prevention programs targeting the general public, such as enlightenment and gatekeeper training programs, were more cost-effective. However, intervention programs for individuals at risk of suicide, which need to utilize collaborations among professionals, such as lawyers, social workers, public health nurses, and psychiatrists, did not contribute to decreasing SMRs [13]. Developing novel suicide prevention programs for socially vulnerable groups requires much more effort than establishing conventional programs for major suicide risk groups. This is due to the planning of various small-scale specific programs that are dependent on the vulnerabilities of each group. Therefore, the development of new programs may lead to reduced efforts in maintaining and promoting established programs, which possibly result in the attenuation of the reduction in SDRs and SMRs.

It has already been reported that the decreasing trends among employed SMRs of both sexes were attenuated after the enactment of the “Work Style Reform Program” in 2018 [54]. The “Work Style Reform Act” contributed to decreasing all types of working hours, including scheduled and non-scheduled working hours of both regular and non-regular employees [54], resulting in successfully controlling the heavy workload, which is an established risk for suicide [55,56]. However, unexpectedly, the wages of non-regular employees were reduced after implementing the “Work Style Reform Act” [54]. Decreasing wages were related to increasing SMRs in non-regular employees [25,57]. Considering the fact that the majority of part-time employees in Japan are female [25], attenuated increasing trends in the wages of part-time employees after enacting the “Work Style Reform Act” might negatively affect some vulnerable female groups. Furthermore, wages of non-regular employees sharply decreased, synchronizing with the pandemic outbreak [54]. Therefore, wage suppression caused by the “Work Style Reform Act” combined with drastically decreasing wages following the onset of the pandemic outbreak may be, at least partially, involved in the sharply increasing SDRs and SMRs of employed females synchronized with the COVID-19 pandemic outbreak.

### 4.2. Impacts of COVID-19 Pandemic

Contrary to GPSPP periods, this study demonstrates that female SDR/SMRs may have been more sensitive to the COVID-19 pandemic, while male SDR/SMRs appear to have been relatively less sensitive to the COVID-19 pandemic. Sex-dependent features, higher susceptibility, and prolonged increasing female SMRs responding to large-scale social shocks were also observed in the Kobe Earthquake (1995) and the Great East Japan Earthquake (2011) [58,59]. In contrast, it has been established that male suicide is more sensitive to economic recession than female suicide [60,61,62]. During the Asian Financial Crisis (1997–1998), Japan experienced drastically increasing male SMRs. In 1997, the male SMR was 26.6 compared to 12.4 in females, and in 1998, the male SMR was 37.1 compared to 15.3 in females [60,63,64]. Considering these previous findings, the features of temporal fluctuation of female SMRs, which sharply increased in synchrony with the COVID-19 pandemic outbreak and subsequently maintained an increase during the pandemic, are similar to the reaction to large-scale disasters rather than economic recession. In other words, the sharply increasing female SDR/SMRs synchronized with the pandemic outbreak are likely to have been induced by psychosocial deterioration rather than socioeconomic deterioration. Indeed, a previous report showed that complete unemployment rates had positive fixed effects on the SMRs of both males and females before the pandemic, whereas during the pandemic, these positive fixed effects on male SMRs were continuously observed, but their effect on female SMRs could not be detected [23,25]. Additionally, different fluctuation patterns between the SMRs of single-person (recovered/decreased) and multiple-person household residents (unchanged) during the pandemic suggest that the transformed lifestyles of individuals due to social restrictions (changes in contact means with individuals outside their household or increasing opportunities for contact with family members) had larger impacts on multiple-person households rather than single-person household residents [27]. Therefore, the specific features of the pandemic compared to earthquakes were that the pandemic forced sustained lifestyle transformations on the entire population.

### 4.3. Impacts ofAENROP and EPU

The results of this study showed interesting commonalities between subgroups that indicated a sharp increase in synchrony with the pandemic outbreak and were insensitive to AENROP, with the exception of unemployed individuals. The SMRs of employed and multiple-person/single-person household females were insensitive to AENROP and sharply increased in synchrony with the pandemic outbreak; however, the SMRs of employed and multiple-person/single-person household males were positively sensitive to AENROP but did not sharply increase. On the contrary, the SMRs of unemployed males and females were insensitive to EPU, but they drastically increased during the pandemic without significantly changing in synchrony with the pandemic outbreak. It has been reported that increasing EPU may have led to increasing suicides in several countries, including the United States, the United Kingdom, and Japan [39,40,41,42,43]. Increasing EPU, which elevates the likelihood of becoming unemployed, may also be reported to be a larger contribution to increasing suicides rather than actually increasing unemployment rates [41]. However, these relationships between EPU and suicides were observed in working-age males but not in females in the United States and Japan [41,43]. Therefore, the sex-dependent responses of SMRs to EPU in Japan detected in this study are similar to findings in previous studies. Another study on the relationship between EPU and suicide suggested that the threat of losing one’s job and the associated financial insecurity is a more powerful risk factor for suicide than actual unemployment [39]. Indeed, although unemployment is a well-known risk for suicide [60,65,66,67,68], several recent studies suggest that the positive impacts of unemployment rates on suicides have been attenuated to the point that they are no longer evaluated as major risk factors in high-income countries, including Japan, due to the favorable effects of generous unemployment insurance programs [24,25,40,41]. In Japan, males are still the primary financial earners in households; however, the vast majority of females are supplementary household earners and non-regular employees (part-timers) [28,29,69] due to the traditional governmental social welfare systems in which governmental social welfare provides generous support systems for households rather than individuals through tax exemptions, universal public pensions, and health insurance systems [69,70,71]. These Japanese household situations can provide insight into why economic uncertainty impacts male suicides but not female suicides [72].

Although the EPU insensitivity of unemployed SMRs is considered to be a rational result, given that unemployed individuals have already lost their jobs, the increase in SMRs among unemployed individuals was severe, approximately five times higher than that just before the pandemic. Political measures to prevent increasing unemployment and employment protection contribute to decreasing SMRs [73,74,75,76,77,78]; however, the impact of supportive measures for unemployed individuals on SMRs cannot necessarily decrease SMRs [76]. SMRs in the region affected by the Great East Japan Earthquake decreased during the recovery phase, but years after, the risk of suicide among lower socioeconomic individuals increased [79]. Therefore, it should be taken into account that not all individuals can benefit from the end of the pandemic or relief from socioeconomic or psychosocial deterioration.

Our hypothesis is supported by the positive relationship between male SDR/SMRs and AENROP, which suggests that increasing government management instability contributes to increasing male SDR/SMRs. However, our hypothesis is largely inconsistent with the lack of sensitivity of female SDR/SMRs to AENROP/EPU, assuming that the increase in female SDR/SMRs in synchrony with the pandemic is due to social shocks. Careful consideration is needed to interpret the sex-dependent discrepancy underlying the causalities between AENROP sensitivity and sharply increasing SDR/SMRs. Based on reports that females show greater affective intensity and more frequently experience negative emotions, such as fear and anxiety, it was reported that females had a greater perception of the severity of the COVID-19 pandemic [80]. It can be easily understood that the pandemic had a greater impact on individuals than political uncertainty (AENROP/EPU). Moreover, the presence of sex-based disparities in political knowledge has historically been established [81,82]. During the pandemic, the government disseminated information about social restriction measures to suppress the expansion of the pandemic, as well as protective countermeasures to support individuals. Taken together, these findings suggest that the replacement of AENROP-sensitivity by susceptibility to political knowledge/information may plausibly explain the differences in the responses to government policies between males and females. In other words, males responded sensitively to governmental protective countermeasures in accordance with government intentions, while female reactions were possibly more sensitive to social shock than government countermeasure information. The positive impacts of AENROP on male SDR/SMRs were more dominant than the impacts of EUP in Japan. In contrast, globally, EPU is reported to be the stronger contributor to increasing SMRs than government uncertainty [39]. The increasing economic uncertainty predominantly contributed to increasing suicide in working-aged males and females over 65 years of age in the United States [41]. Therefore, the sensitivities of males in Japan and the United States to EPU are partially similar, whereas those of females were not identical. In other words, the dominant impacts of AENROP on SDRs/SMRs disaggregated by social standing in Japan may not be generalizable to other countries due to the possibly specific situation in Japan.

Other than suicide, uncertainty has been reported to be related to mortalities caused by cardiovascular disease and traffic accidents in the United Kingdom and the United States [83,84,85]. Furthermore, increasing uncertainty contributed to deteriorating well-being and mental health in the United Kingdom around the Brexit period and the United States aftermath of the Trump election [50,86,87]. Notably, antidepressant prescribing began to increase before the Brexit referendum in 2016 but became slower after the referendum [87]. Therefore, stabilizing the government and its political implementation plays important roles in controlling not only suicide mortality but also protection for mental health. Considering these findings, several types of psychological/psychiatric therapies/medications may improve well-being or prevent suicides of some individuals. However, the mass media must consider the possibility that reporting criticism about government policies without explanation regarding the purpose and rationale of policies, thereby increasing anxiety among the public (viewers), results in deteriorating mental health in Japan.

### 4.4. Limitations

This study is associated with several limitations. The EPU index for Japan is obtained through text mining techniques from four major newspapers, based on the method of Baker et al. (2016) [47,49]. AENROP is derived from public opinion polls that are regularly conducted by newspapers, news agencies, and TV networks [47]. Recently, it has become well-known that social media contributes to shaping opinions on societal problems [88]. However, the EPU and AENROP are not derived from social media information. The impacts of the uncertainty indices detected in this study may, therefore, differ slightly from recent situations.

When analyzing the impact of uncertainty, it is important to add independent variables that can reflect reality, such as unemployment rates, GDP per capita, and household incomes. However, most published data are based on pre-pandemic values, and values that have been published have been quarterly values. Therefore, to implement a detailed analysis of the temporal causalities between monthly SDRs and SMRs and uncertainty indices during the pandemic, we must wait for the government to release the 2020–2023 data.

This study suggests that both government economic policy uncertainty and government management instability contribute to increasing suicides, likely anomic suicides. Therefore, stabilizing the government and its political implementation can play an important role in controlling suicide mortality. Therefore, several types of psychological/psychiatric therapies/medications may provide improved well-being or prevent suicide in some individuals. To explore these possibilities, further studies are needed.

## 5. Conclusions

This study aimed to determine the fluctuations in SDRs and SMRs disaggregated by sex and social standing during three GPSPP periods and the COVID-19 pandemic outbreak and causality from uncertainty indices (EPU and AENROP) to SDRs and SMRs from January 2009 to June 2023, in order to clarify the causes of recently increasing suicides in Japan. The GPSPP contributed to decreasing SDR/SMRs during socially stable periods without large-scale disasters, economic shocks, or drastically changing social systems since decreasing trends in all SDRs and SMRs, except for unemployed females in the second GPSPP, were observed. On the contrary, decreasing trends in these SDR/SMRs were already attenuated before the pandemic in the third GPSPP. Therefore, the attenuation of decreasing trends in SDR/SMRs in the early phase of the third GPSPP was possibly independent of the pandemic. Female SMRs of employed individuals and multiple-person/single-person household residents, which were insensitive to AENROP or EPU, sharply increased in synchrony with the pandemic and subsequently did not recover during the pandemic. In contrast, the male SMRs of employed individuals and multiple-person/single-person household residents, which were positively related to AENROP and EPU, did not sharply increase and did not show any remarkable change after the outbreak. The SMRs of unemployed males (AENROP-sensitive but EPU-insensitive) and females (insensitive to AENROP or EPU) did not increase sharply at the pandemic outbreak, whereas they increased drastically in the late of the pandemic. Therefore, although increasing AENROP contributed to increasing male SDRs and SMRs during the entire observation period, the appropriate policy announcements could contribute to suppressing the largely or rapidly increasing SDRs and SMRs of males during large-scale social shocks in Japan. On the contrary, the groups with insensitivity to AENROP may be predominantly dominated by large-scale social shocks compared to the governmental policy containment efforts, resulting in an inability to consequently suppress increasing suicides. The sensitivity to AENROP suggests that AENROP is an indicator of not only political uncertainty but also of individuals’ susceptibility to political knowledge and their affinity for political information.

## Figures and Tables

**Table 1:** Descriptive statistics of average population and suicides from January 2009 to June 2023 in Japan.

	Males + Females		Males		Females	
(population)	*Mean*	*SD*	*Mean*	*SD*	*Mean*	*SD*
total population	127,268	885	62,103	420	65,165	469
multiple-person household residents	108,051	2475	52,726	1222	55,325	1254
single-person household residents	19,217	1926	9377	936	9840	990
employed individuals	65,207	1974	36,804	432	28,404	1583
unemployed individuals	2239	605	1351	389	888	217
(suicide numbers)						
total population	355,746		245,384		110,507	
multiple-person household residents	237,965		156,918		81,119	
single-person household residents	106,961		80,425		26,551	
employed individuals	128,155		106,534		21,617	
unemployed individuals	16,313		14,502		1839	

*SD*: standard deviation.

**Table 2:** Fixed effects of EPU indices on SDR/SMR.

SDRs											
Males			*F* Value	*p* Value			*β*	*SE*	*Z* Value	*p* Value	
*F*(46,	2827.9)	=	24.429	0.000	**	EPU	0.032	0.015	2.100	0.041	*
LSDV	*R* ^2^	=	0.125								
Within	*R* ^2^	=	0.010								
females											
*F*(46,	2828.1)	=	0.522	0.997		EPU	0.010	0.006	1.611	0.114	
LSDV	*R* ^2^	=	0.006								
Within	*R* ^2^	=	0.003								
**MaleSMRs**											
employed											
*F*(46,	2827.5)	=	25.339	0.000	**	EPU	0.022	0.003	8.509	0.000	**
LSDV	*R* ^2^	=	0.112								
Within	*R* ^2^	=	0.004								
unemployed											
*F*(46,	2826.2)	=	16.774	0.000	**	EPU	0.114	0.065	1.759	0.085	
LSDV	*R* ^2^	=	0.055								
Within	*R* ^2^	=	0.001								
multiple-person											
*F*(46,	2827.6)	=	37.174	0.000	**	EPU	0.026	0.003	10.290	0.000	**
LSDV	*R* ^2^	=	0.160								
Within	*R* ^2^	=	0.008								
single-person											
*F*(46,	2827.5)	=	21.684	0.000	**	EPU	0.079	0.036	2.159	0.036	*
LSDV	*R* ^2^	=	0.067								
Within	*R* ^2^	=	0.005								
**Female SMRs**											
employed											
*F*(46,	2828.1)	=	0.765	0.875		EPU	0.002	0.003	0.535	0.595	
LSDV	*R* ^2^	=	0.004								
Within	*R* ^2^	=	0.000								
unemployed											
*F*(46,	2828.0)	=	0.463	0.999		EPU	0.027	0.025	1.090	0.281	
LSDV	*R* ^2^	=	0.003								
Within	*R* ^2^	=	0.000								
multiple-person											
*F*(46,	2828.1)	=	0.759	0.882		EPU	0.009	0.005	1.671	0.102	
LSDV	*R* ^2^	=	0.007								
Within	*R* ^2^	=	0.003								
single-person											
*F*(46,	2828.1)	=	0.780	0.857		EPU	0.012	0.011	1.045	0.301	
LSDV	*R* ^2^	=	0.005								
Within	*R* ^2^	=	0.000								

LSDV: least square dummy variables, *SE*: standard error, *β*: coefficient value, SDR: age-standardized suicide death rate per 100,000 in the population, SMR: standardized suicide mortality per 100,000 population, * *p* < 0.05, and ** *p* < 0.01.

**Table 3:** Fixed effects of AENROP and EPU indices on SDRs/SMRs (HLM).

MaleSDR	*F* Value	*p* Value			*β*	*SE*	*Z* Value	*p* Value	
*F*(46, 2827.8)	29.573	0.000	**	AENROP	0.094	0.011	8.489	0.000	**
LSDV *R*^2^	0.231			EPU	0.004	0.013	0.326	0.744	
Within *R*^2^	0.129								
**Female SDR**									
*F*(46, 2828.1)	0.565	0.992		AENROP	0.039	0.004	9.296	0.000	**
LSDV *R*^2^	0.078			EPU	−0.002	0.005	−0.383	0.702	
Within *R*^2^	0.076								
**Male SMRs**									
**employed**									
*F*(46, 2827.4)	28.791	0.000	**	AENROP	0.075	0.010	7.860	0.000	**
LSDV *R*^2^	0.169			EPU	0.000	0.011	0.000	1.000	
Within *R*^2^	0.068								
**unemployed**									
*F*(46, 2826.2)	17.014	0.000	**	AENROP	0.232	0.053	4.406	0.000	**
LSDV *R*^2^	0.061			EPU	0.045	0.063	0.711	0.477	
Within *R*^2^	0.007								
**multiple-person**									
*F*(46, 2827.5)	45.404	0.000	**	AENROP	0.081	0.009	8.711	0.000	**
LSDV *R*^2^	0.250			EPU	0.002	0.011	0.146	0.884	
Within *R*^2^	0.115								
**single-person**									
*F*(46, 2827.3)	25.430	0.000	**	AENROP	0.205	0.027	7.516	0.000	**
LSDV *R*^2^	0.105			EPU	0.018	0.033	0.554	0.579	
Within *R*^2^	0.046								
**Female SMRs**									
**employed**									
F(46, 2828.1)	0.779	0.858		AENROP	0.016	0.002	6.471	0.000	**
LSDV *R*^2^	0.017			EPU	−0.003	0.003	−1.019	0.308	
Within *R*^2^	0.013								
**unemployed**									
*F*(46, 2828.0)	0.462	0.999		AENROP	0.012	0.021	0.554	0.579	**
LSDV *R*^2^	0.003			EPU	0.024	0.026	0.921	0.357	
Within *R*^2^	0.000								
**multiple-person**									
*F*(46, 2828.1)	0.814	0.810		AENROP	0.036	0.004	9.369	0.000	**
LSDV *R*^2^	0.070			EPU	−0.001	0.005	−0.326	0.745	
Within *R*^2^	0.066								
**single-person**									
*F*(46, 2828.1)	0.792	0.841		AENROP	0.056	0.009	6.490	0.000	**
LSDV *R*^2^	0.017			EPU	−0.005	0.010	−0.472	0.637	
Within *R*^2^	0.013								

LSDV: least square dummy variables, *SE*: standard error, *β*: coefficient value, SDR: age-standardized suicide death rate per 100,000 of the population, SMR: standardized suicide mortality per 100,000 population, AENROP: government management instability, EPU: economic policy uncertainty ** *p* < 0.01.

**Table 4:** Temporal causality from AENROP and EPU to SDRs/SMRs (VAR).

	Adjusted *R*^2^	*F*(3169)	*p* Value			*β*	*SE*	*T* Value	*p* Value	
Males + Females	0.854	227.53	0.0001	**	SDR	0.866	0.038	22.540	0.000	**
					AENROP	0.987	0.407	2.423	0.017	*
					EPU	−0.134	0.467	−0.288	0.774	
males	0.865	234.45	0.0001	**	SDR	0.868	0.038	22.670	0.000	**
					AENROP	1.537	0.562	2.735	0.007	**
					EPU	−0.339	0.690	−0.492	0.624	
females	0.750	141.45	0.0000	**	SDR	0.797	0.052	15.290	0.000	**
					AENROP	0.685	0.358	1.915	0.057	
					EPU	0.179	0.360	0.496	0.621	
**Males**										
employed	0.803	149.41	0.0001	**	employed	0.803	0.047	17.260	0.000	**
					AENROP	1.738	0.507	3.430	0.001	**
					EPU	−0.513	0.617	−0.831	0.407	
unemployed	0.582	52.46	0.0001	**	unemployed	0.705	0.064	10.990	0.000	**
					AENROP	7.569	3.104	2.438	0.016	*
					EPU	−2.588	4.300	−0.602	0.548	
multiple-person	0.872	245.27	0.0001	**	multiple-person	0.872	0.038	23.150	0.000	**
					AENROP	1.216	0.417	2.915	0.004	**
					EPU	−0.402	0.552	−0.728	0.468	
single-person	0.843	218.81	0.0001	**	single-person	0.860	0.041	21.090	0.000	**
					AENROP	5.138	2.227	2.307	0.022	*
					EPU	−0.535	2.433	−0.220	0.826	
**Females**										
employed	0.479	69.97	0.0000	**	employed	0.628	0.060	10.450	0.000	**
					AENROP	0.406	0.235	1.731	0.085	
					EPU	0.113	0.271	0.419	0.676	
unemployed	0.404	31.73	0.0000	**	unemployed	0.639	0.067	9.473	0.000	**
					AENROP	0.938	1.128	0.832	0.407	
					EPU	−1.445	1.756	−0.823	0.412	
multiple-person	0.765	162.64	0.0000	**	multiple-person	0.807	0.049	16.380	0.000	**
					AENROP	0.610	0.314	1.942	0.054	
					EPU	0.107	0.317	0.336	0.737	
single-person	0.642	77.13	0.0000	**	single-person	0.743	0.059	12.510	0.000	**
					AENROP	1.675	0.855	1.959	0.052	
					EPU	0.053	1.130	0.047	0.963	

*SE*: standard error, *β*: coefficient value, SDR: age-standardized suicide death rate per 100,000 of the population, SMR: standardized suicide mortality per 100,000 population, AENROP: government management instability, EPU: economic policy uncertainty * *p* < 0.05, and ** *p* < 0.01.

## Data Availability

Raw data are publicly available to any persons via Japanese national databases from the “Basic Data on Suicide in the Region” (BDSR) in the MHLW, the “Regional Statistics of the System of Social and Demographic” in the e-Stat, and the indices of EPU and AENROP in the database of RIETI.

## Supplementary Materials

The following supporting information can be downloaded at https://www.mdpi.com/article/10.3390/ijerph21101366/s1. Table S1. Priority major categories of governmental “General Principles of Suicide; Table S2. Term Sets for EPU index [47].
