# Supplementary material for: Uncertainties of Economic Policy and Government Management Stability Played Important Roles in Increasing Suicides in Japan from 2009 to 2023
Source: Int J Environ Res Public Health. 2024 Oct 16;21(10):1366. doi: 10.3390/ijerph21101366 (PMC11507343; doi:10.3390/ijerph21101366)
Supplement: Supplementary file 1 [file ijerph-21-01366-s001.zip › ijerph-3235823-supplementary.pdf]

Supplementary Data List

Supplementary Table s1: Priority major categories of governmental "General Principles of Suicide Prevention Policy" (GPSPP) in 2nd (August-2012 to July-2017) and 3rd (July-2017 to October-2022) periods (p2-4)

Supplementary Table s2: Term Sets for EPU index (p6)

Supplementary Table s1: Priority major categories of governmental "General Principles of Suicide Prevention Policy" (GPSPP) in 2nd (August-2012 to July-2017) and 3rd (July-2017 to October-2022) periods

2nd GPSPP (August-2012 to July-2017)

1.Clarify the actual situation of the suicide
Investigation of the actual situation of suicide
Enhancement of information of reporting system
Survey about the suicide prevention of student
Investigations of pathophysiology of mood disorder

2. Promoting awareness and observation by each individual
Enlightenment of suicide prevention programme week
Implementation of education about suicide prevention among students
Enlightenment of mood disorder
Dissemination of knowledge about suicide and suicide-related events

3. Development of Professionals for suicide prevention
Psychiatric education for general physicians
Enlightenment of suicide and suicide prevention programme to educationists
Implementation of training for long-term care support specialists
Improving the quality of counsellors related to overloaded debts, unemployment and economic hardship
Development of gatekeeper

4. Promoting mental health
Promotion of occupational mental health supports
Development of mental health support system in community
Development of mental health support service in schools
Enhancement of mental care for victims of disasters and support their life reconstruction

5. Enhancement of psychiatric care system
Development of psychiatric co-medical staffs
Increasing consultation rate of depression
Enhancement of measures for high-risk individuals with mental illnesses other than depression
Support for patients with chronic diseases

6. Preventing suicide through social cooperation
Enhancement of consultation system in community
Enhancement of consultation system for overloaded debts
Enhancement of counselling system for unemployed people
Enhanced support for caregivers
Disseminate WHO guideline to the mass media
Preventing suicide in bullied children
Enhancement of for telephone counselling service for bullying children
Preventing threats of suicide in Internet
Responding to suicide notice using Internet
Enhancement of support systems for victims of child abuse and sex crime
Enhancement of support system for economic hardship

7. Preventing repeated suicidal behaviours in suicide attempters
Improving the liaison consultation psychiatric system in medical facilities
Support for observation/protection by family members

8. Enhancement of support for bereaved families of suicide victim
Supporting the self-help groups for bereaved families of suicide victims

Development of supporting systems for bereaved families of suicide victim
Development of brochure for bereaved families of suicide victim

9. Enhancement of cooperation with private organizations
Development of human resource for suicide prevention
Establishment of regional cooperation system
Support for telephone counselling service of private organizations
Support for pioneering/trial efforts by private organizations

Blue painted lists were improved priority categories from 1st to 2nd GPSPP.

3rd GPSPP (July-2017 to October-2022)

1. Clarify the actual situation of the suicide
Innovative suicide research promotion programme
(investigative research, verification and utilization of survey findings)
Collection, organization and provision of information on advanced initiatives
Survey of children/adolescent suicides
Coordination with the cause-of-death investigation system
Accumulation, organization and analysis of information related to suicide prevention

2. Promoting awareness and observation by each individual
Enlightenment of suicide prevention programme week
Implementation of education about suicide prevention among student
Enlightenment of mood disorder
Dissemination of knowledge about suicide and suicide-related events
Enlightenment of importance about SOS

3. Development of Professionals for suicide prevention
Psychiatric education for general physicians
Improving the quality of community/occupational health staffs
Development of gatekeepers
Support for supporters including family members and acquaintances
Promotion of education of suicide prevention in university

4. Promoting mental health
Promotion of occupational mental health supports
Development of mental health support system in community
Development of mental health support service in schools
Enhancement of mental care for victims of disasters and support their life reconstruction

5. Enhancement of psychiatric care system
Enhancement of measures for high-risk individuals with mental illnesses other than depression
Enhancement of measures for high-risk individuals with mental illnesses, including depression, schizophrenia, several dependencies
Development of human resources responsible for mental health medical welfare services

6. Preventing suicide through social cooperation
Enhancement of consultation system in community
Enhancement of consultation system for overloaded debts
Enhancement of counselling system for unemployed people
Enhanced support for caregivers
Disseminate WHO guideline to the mass media
Preventing suicide in bullied children
Enhancement of for telephone counselling service for bullying children
Preventing threats of suicide in Internet
Responding to suicide notice using Internet
Enhancement of support systems for victims of child abuse and sex crime

Enhancement of support system for economic hardship
Enhancement of consultation services using internet and SNS
Development of diverse consultations and strengthening outreach
Disseminate information sharing necessary for cooperation among related organizations
Promoting of places for stay contributing to suicide prevention
Enhancement of support for expectant and nursing mothers
Enhancement of support for LGBT

7. Preventing repeated suicidal behaviours in suicide attempters
Enhancement of comprehensive support systems for suicidal attempters via collaboration among medical and community
Development of regional medical centre for prevention repeated suicide attempt behaviours
Development of safety places
Development of supporting systems in schools and workplaces

8. Enhancement of support for bereaved families of suicide victim
Supporting the self-help groups for bereaved families of suicide victims
Development of brochure for bereaved families of suicide victim
Enhancement of support for bereaved children of suicide victim
Improving the quality of governmental staffs contact with bereaved families
Enhancement of provision of information for comprehensive support bereaved families of suicide victims
Development of supporting systems for bereaved families of suicide victims in schools and workplaces

9. Enhancement of cooperation with private organizations
Development of human resource for suicide prevention
Establishment of regional cooperation system
Support for telephone counselling service of private organizations
Support for pioneering/trial efforts by private organizations

10. Development of suicide prevention programme for child/adolescent
Enhancement of suicide prevention of suicide among bullying children
Enhancement of support for students
Education how to request supports
Enhancement of support system for children
Enhancement of support system for adolescents
Enhancement of support young generation based on their specific features
Support for acquaintances

11. Enhancement of prevention of suicide caused by employment-related causes
Enhancement of long working hours promotion of mental health in workplace
Harassment prevention measures

12. Enhancement of regional suicide prevention programmes
Development of regional suicidal profile and political package for regional suicide prevention programmes
Development of guidelines for regional suicide prevention programmes
Enhancement of regional suicide prevention centres
Promoting the establishment of dedicated departments and professional staffs for suicide prevention programmes in regional governments

---

Blue painted lists were newly added priority categories from 2nd to 3rd GPSPP.

Supplementary Table s2:

Term Sets for EPU index[1]

Economy terms	
	"economic" or "economy"
	Uncertainty terms
	"uncertain" or "uncertainty"
	"concern"
Policy terms	
	"tax(es)"
	"taxation"
	"government spending" or "government expenditure"
	"government revenue(s)"
	"government budget"
	"public debt"
	"government debt"
	"government deficit(s)"
	"BOJ"
	"Bank of Japan"
	"central bank(s)"
	"The Fed"
	"Federal Reserve"
	"regulation(s)","regulatory","regulate", "deregulation" or "deregulate"
	"structural reform"
	"legislation"
	"upper house"
	"lower house"
	"Diet"
	"Prime minister"
	"Prime minister's office"

1. RIETI. Japan Economic Policy Uncertainty Index. Available online: https://www.rieti.go.jp/jp/database/policyuncertainty/index.html (accessed on 2023.12.1).
